# Wind Tunnel Tests for Wind Pressure Distribution on Gable Roof Buildings

**DOI:** 10.1155/2013/396936

**Published:** 2013-09-03

**Authors:** Xiao-kun Jing, Yuan-qi Li

**Affiliations:** ^1^Department of Building Engineering, Tongji University, Shanghai 200092, China; ^2^State Key Laboratory for Disaster Reduction in Civil Engineering, Tongji University, Shanghai 200092, China

## Abstract

Gable roof buildings are widely used in industrial buildings. Based on wind tunnel tests with rigid models, wind pressure distributions on gable roof buildings with different aspect ratios were measured simultaneously. Some characteristics of the measured wind pressure field on the surfaces of the models were analyzed, including mean wind pressure, fluctuating wind pressure, peak negative wind pressure, and characteristics of proper orthogonal decomposition results of the measured wind pressure field. The results show that extremely high local suctions often occur in the leading edges of longitudinal wall and windward roof, roof corner, and roof ridge which are the severe damaged locations under strong wind. The aspect ratio of building has a certain effect on the mean wind pressure coefficients, and the effect relates to wind attack angle. Compared with experimental results, the region division of roof corner and roof ridge from AIJ2004 is more reasonable than those from CECS102:2002 and MBMA2006.The contributions of the first several eigenvectors to the overall wind pressure distributions become much bigger. The investigation can offer some basic understanding for estimating wind load distribution on gable roof buildings and facilitate wind-resistant design of cladding components and their connections considering wind load path.

## 1. Introduction

Light-weight buildings with gable roof have been widely used in low-rise industrial buildings due to high degree of industrialization and rapid speed of construction; however, their relatively light dead load and flexibility make them most vulnerable to wind load which becomes a very important load type in structural design. In recent years, light-weight buildings with gable roof were usually damaged severely under strong wind, which caused huge economic losses.

An accurate knowledge of wind pressure or their coefficients on the walls and roofs of buildings is essential to the wind-resistant design of structures. Holmes [1] investigated the characteristics of wind pressure acting on the walls and roofs of gable-roofed tropical houses considering the effects of elevation, roof pitch, and grouping of buildings on the external pressures. Kanda and Maruta [2] investigated the characteristics of long low-rise building with gable roof for the case of large aspect ratio. Case and Isyumov [3] showed that the suburban exposure produces lower wind loads than those experienced in the open country exposure by the comparisons of local pressures and selected structural loads on low buildings with 4 : 12 gable roof.

It is well known that the structural systems of such light-weight buildings with gable roof consist of portal or pin-jointed frames. The metal sheathings are attached to the roof purlins and the wall girts by the fasteners, which are fixed to these frames. Wind load path has significant effect on the effective wind loaded area of cladding components which are definitely not mentioned in current wind-resistant design, especially for the fasteners. The long-term objective of this study is to investigate how actual wind load is equivalent to extreme wind load on main structure, cladding components, and fasteners of light-weight steel structure considering the spatial correlation of wind pressure and wind load path in wind-resistant design.

The present study, however, is only a preliminary study on gable roof buildings with emphasis on the understanding of wind pressure on gable roof buildings, through comparisons with existing results and relative wind load codes, to facilitate wind-resistant design of cladding components and their connections considering wind load path.

## 2. Experimental Technique

The wind tunnel tests were carried out in the Boundary Layer Wind Tunnel at State Key Laboratory for Disaster Reduction in Civil Engineering at Tongji University. This wind tunnel has a working section of 15 m length, 3 m width, and 2.5 m height.

A 1 : 40 scale model of natural wind was developed in the wind tunnel to simulate the natural wind over terrain category B defined in *Load Code for the Design of Building Structures* (GB50009-2001) of China [4] by using spires and an array of blocks on the tunnel floor. The mean wind speed profile measured in the tunnel was in good agreement with the power law of an exponent of 0.16. The mean wind speed was 8 m/s, and the longitudinal turbulence was 0.22 measured at the mean roof heights of the building models which are taken as the reference heights in analyzing pressure coefficients. It should be pointed out that the wind tunnel tests were completed before *Load Code for the Design of Building Structures* (GB50009-2012) of China [5] was published. In GB50009-2012, the exponent of power law of the mean wind speed profile in terrain category B is 0.15 instead of 0.16, which properly decreases mean wind load in terrain category B. It has almost no effect on the analysis of characteristics of wind pressure in this paper.

**Figure 1:**
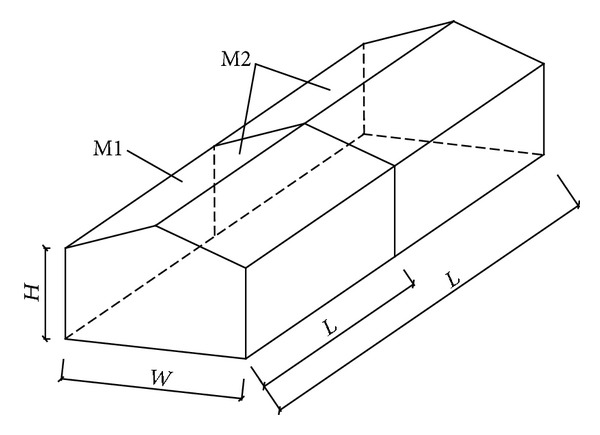
Gable roof buildings.

**Figure 2:**
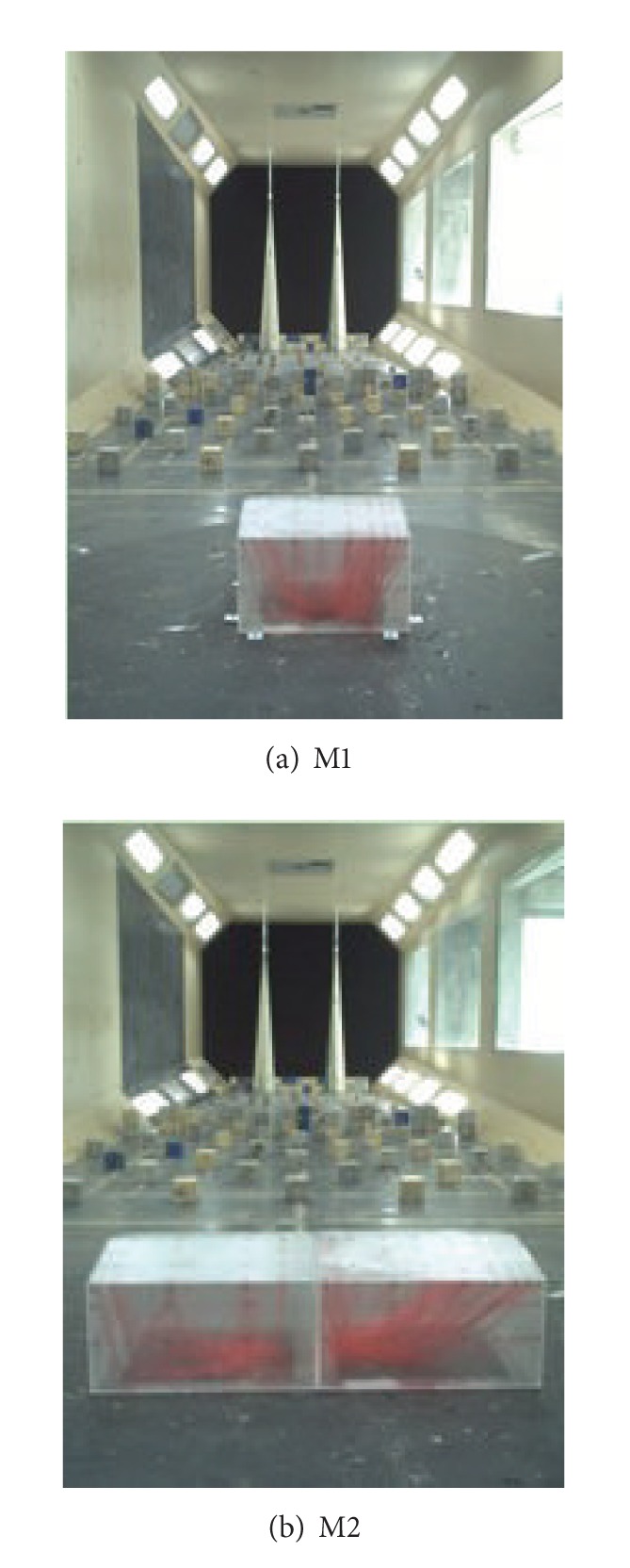
Pictures of M1 and M2 in wind tunnel tests.

The prototype buildings are of two different lengths with constant width, height, and roof pitch, as shown in Figure 1. *H*, *W*, and *L* represent the eave height, width, and length of building, respectively.  Table 1 summarizes the building dimensions. For a convenient description, the gable roof building model with length to width aspect ratios (*L*/*W*) 1.0 is designated as M1, and the gable roof building model with length to width aspect ratios (*L*/*W*) 2.0 is designated as M2. The roof pitches of M1 and M2 are 10°.  Figure 2 shows two pictures of M1 and M2 in wind tunnel tests.

**Figure 3:**
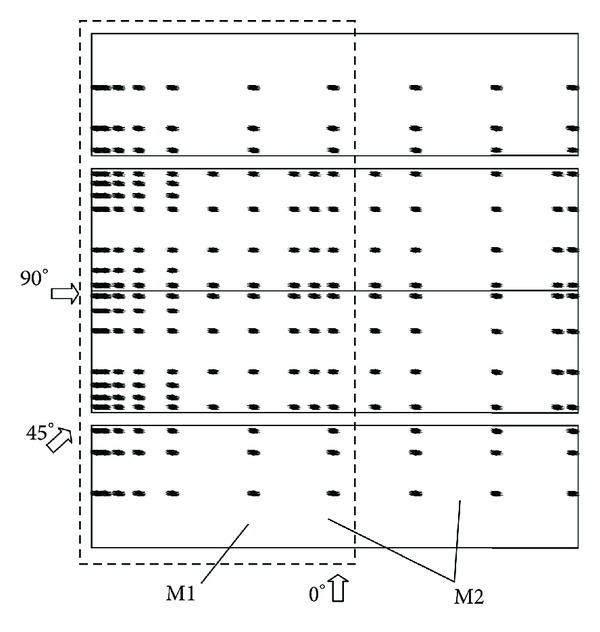
Layout of pressure taps on gable roof building models.

A total of 152 taps for M1 and 210 taps for M2 were arranged on the roofs and longitudinal walls, as shown in Figure 3. Particular attention was paid to the number and positions of the taps near the roof corner, roof edge, and roof ridge. The layout of pressure taps for western half part of M2 is the same as that for M1. Each model was tested at a 45° increment from 0° to 360°.

The pressures at all taps were measured simultaneously and sampled at a sampling rate of 312.5 Hz. For each run wind pressures measured on the models were expressed in the form of a nondimensional pressure coefficient, *C*_*p*_(*t*), defined as follows:

(1) $$\begin{array}{c} C_{p}\left(t\right)=\frac{p\left(t\right)-p_{o}}{0.5\rho U_{}^{2}}, \end{array}$$

where *p*(*t*) is the measured wind pressure on the surface of model, *p*_*o*_ is the static pressure at the reference height, *U* is the mean wind speed at the reference height, and *ρ* is the air density.

The mean pressure coefficient, standard deviation pressure coefficient, and maximum and minimum pressure coefficients were calculated from each wind attack angle. The sign of the wind pressure coefficient indicates the direction of wind pressure on the surface of building model; positive value indicates wind pressure acting towards the surface and negative value indicates wind pressure acting away from the surface (suction).

## 3. Mean Wind Pressure

### 3.1. Experimental Results

**Figure 4:**
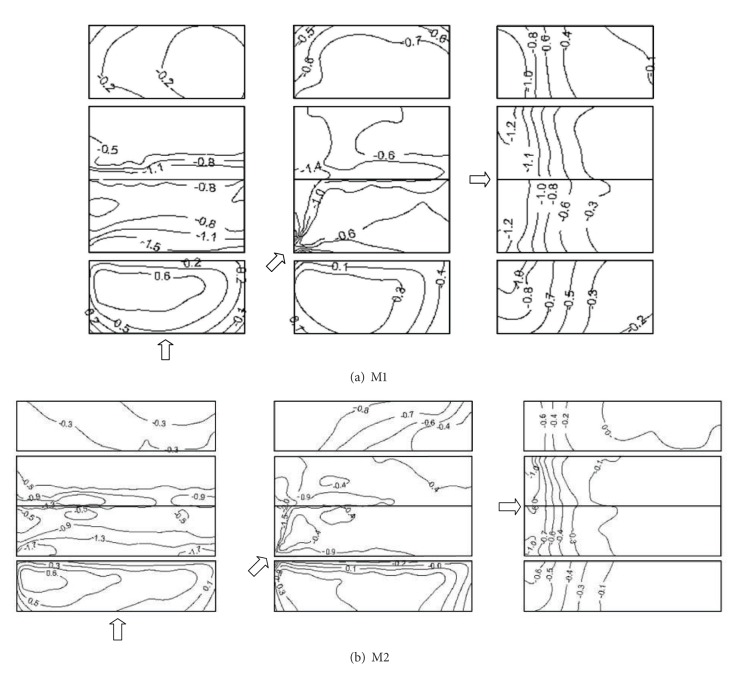
Mean wind pressure coefficients on M1 and M2.

In consideration of the symmetric conditions of the building models, Figure 4 shows the measured results of mean wind pressure coefficients on M1 and M2 with wind attack angles 0°, 45°, and 90°, respectively.

The magnitude and distribution of mean wind pressure coefficients on M1 can be drawn from Figure 4(a). In the 0° wind direction, namely the wind direction normal to the roof ridge, the windward wall experiences positive pressures and higher positive pressure is about 0.6 in the middle of the windward wall. High mean suctions occur on the leading edge of the windward roof and leeward roof ridge with mean wind pressures coefficients of −1.5 (the leading edge) and −1.1 (the leeward roof ridge). The occurrence of these high mean suctions is believed to be caused by the separation bubbles when the air flow is probably separated from the leading edge of the windward roof and the windward roof ridge. The mean suctions are uniformly distributed over the leeward wall, and the corresponding mean wind pressure coefficient is about −0.2. In the 45° wind direction, namely, the wind direction oblique to the roof ridge, the windward wall experiences smaller positive pressure mostly compared with the wind direction of 0°. The formation of conical vortex in two sides of separation region, due to the separation of air flow from the leading edge of the roof, results in higher suction near the roof corner, and the corresponding maximum suction coefficient is up to −1.4. The leeward wall experiences suction between the wind directions of 0° and 90°. In the 90° wind direction, namely, the wind direction parallel to the roof ridge, the windward region of walls experiences high suctions due to the separation of air flow from the leading edges of walls, and the corresponding maximum suction coefficient is up to −1.0. The mean suction coefficients on the longitudinal walls decrease with the increase of the distance to the leading edges of two longitude walls. High suctions appear in the leading edge of the windward roof resulting from the formation of the separation bubble, and the corresponding maximum suction coefficient is up to −1.2. The mean suction coefficients on the roof decrease with the increase of the distance to the leading edge of the roof.

Figure 4(b) shows that, in general, the distribution of mean wind pressure coefficients with the same wind direction on M2 is similar to that on M1. In the 0° wind direction, the mean wind pressure coefficients on the roof and leeward wall of M2 are larger than those of M1. In the 45° wind direction, the mean wind pressure coefficients on the windward wall and roof of M2 are approximate to those of M1. The mean pressure coefficients on the end region of leeward wall of M2 are larger than those of M1, which is in agreement with Kanda and Maruta's results [2] that an increase in the aspect ratio is shown to slightly increase the mean suction on the leeward wall for wind direction of 45°. In the 90° wind direction, the mean pressure coefficients on the walls and roof of M2 are significantly smaller than those of M1. It is concluded that the aspect ratio has a certain effect on mean wind pressure coefficients and the effect relates to wind attack angle.

### 3.2. Comparative Analysis

#### 3.2.1. Comparison with Holmes' Work

**Figure 5:**
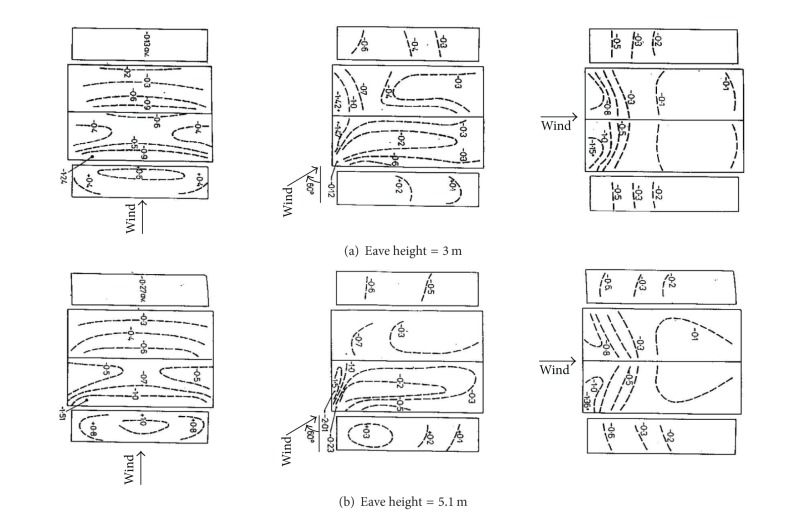
Mean wind pressure coefficients on gable roof buildings with roof pitch 10° (Holmes' results).

Holmes carried out a series of wind tunnel study on wind pressure on gable roof buildings.  Figure 5 shows his results on the magnitude and distribution of mean wind pressure coefficients on gable roof buildings with different eave heights for 0°, 60°, and 90° wind directions, respectively. It can be seen that the roof pressure coefficients are invariably negative for all wind directions, and the walls and roof pressure coefficients are significantly higher for the elevated building [1]. Although Holmes' results give mean wind pressure coefficients for 60° wind direction, the formation of conical vortex still occurs in the small separation region of roof corner and generates high suction, and only the shape of conical vortex is different from 45° wind direction in these wind tunnel tests. The distribution of mean wind pressure coefficients on the walls and roof in these wind tunnel tests is basically consistent with Holmes' results. The eave heights of M1 and M2 are 9 m. Mean pressure coefficients on M1 and M2 are remarkably larger than those inFigure 5(b) of Holmes' results apart from positive pressure on the windward wall for 0° wind direction. It is concluded that mean pressure coefficients on the walls and the roof increase with the increase of building height combined with Holmes' results.

#### 3.2.2. Comparison with TPU's Work

**Figure 6:**
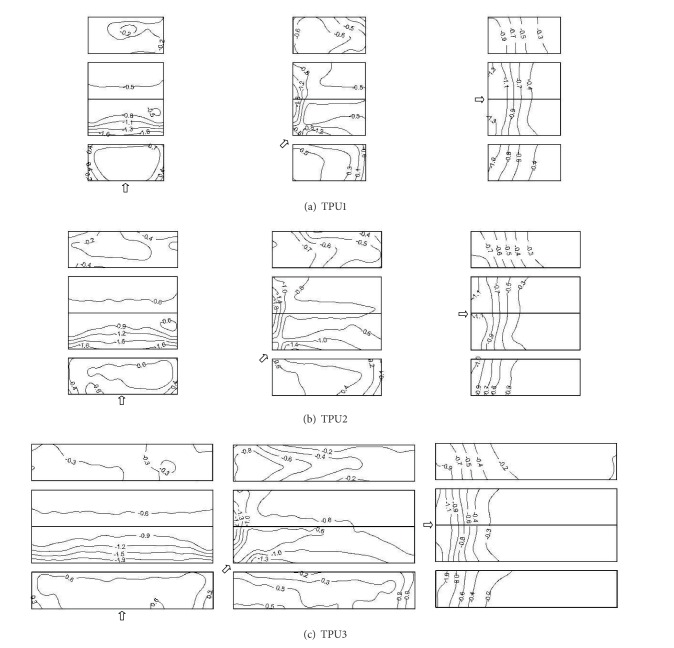
Mean wind pressure coefficients on gable roof buildings with roof pitch 10° (TPU's results).

Tokyo Polytechnic University (TPU) performed comprehensive wind tunnel tests of wind pressure on buildings and established database which have been published on the website, including the characteristics of wind pressure on low-rise buildings [6].  Table 2 shows selected full scale gable roof building dimensions of TPU for comparative analysis. The model scale is 1 : 100. The taps are uniformly arranged on the surfaces of selected TPU models.  Figure 6 shows the magnitude and distribution of mean wind pressure coefficients on selected TPU models.

In the same wind direction, the distributions of mean wind pressure coefficients on selected TPU models are similar and have no variations with the variation of aspect ratio. In the 0° wind direction, there is an increase in mean wind pressure coefficients on the roofs and leeward walls with the increase of the aspect ratio; particularly, the increase in mean wind pressure coefficients on the leading edge of windward roof is significant. In the 45° wind direction, mean wind pressure coefficients on the leeward wall increase as the aspect ratio increases, while mean wind pressure coefficients on the windward wall and roof have mostly no variations. In the 90° wind direction, an increase in the aspect ratio is shown to decrease mean wind pressure coefficients on the walls significantly and roofs slightly. The above TPU's results are similar to the experimental results in this paper.

In the same wind direction, the distributions of mean wind pressure coefficients on M1 and TPU1 are similar, and mean wind pressure coefficients on TPU1 are close to M1. In the same wind direction, the distributions of mean wind pressure coefficients on TPU2, M2, and TPU3 are similar and have no variations with the variation of aspect ratio. In the 0° wind direction, mean wind pressure coefficients on M2 are between TPU2 and TPU3. High local suction and high positive pressure occur on the roof ridge and windward wall of M2. In the 45° wind direction, mean wind pressure coefficients on TPU2, M2, and TPU3 are very close; however suctions caused by the formation of conical vortex on M2 are a little larger than those on TPU2 and TPU3 due to the different turbulence intensities. In the 90° wind direction, mean wind pressure coefficients on M2 are between TPU2 and  TPU3. Suctions on the leading edge of windward roof of M2 are smaller, which is in accordance with the laws that state that mean wind pressure coefficients on the longitudinal walls and roof decrease as the aspect ratio increases.

## 4. Fluctuating Wind Pressure

**Figure 7:**
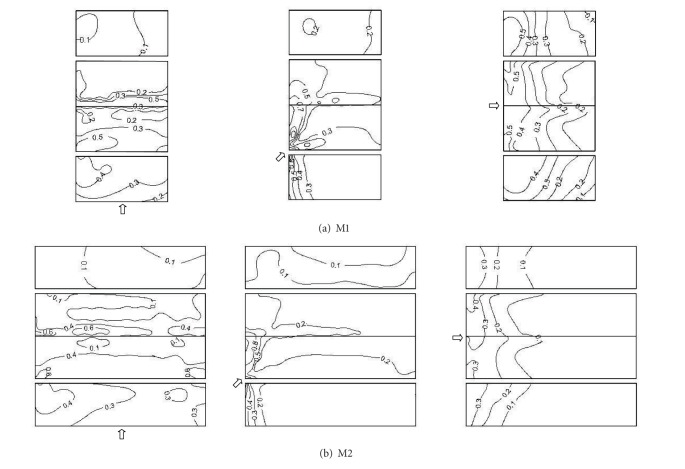
Fluctuating wind pressure coefficients on M1 and M2.

The fluctuating level of wind pressure occurring at a particular point and for a particular wind direction can be estimated by the standard deviation pressure coefficient. In consideration of the symmetric conditions of the building models, Figure 7 shows the measured results of fluctuating wind pressure coefficients on M1 and M2 with wind attack angles 0°, 45°, and 90°, respectively.

Figure 7(a) shows the distributions of fluctuating wind pressure coefficient are similar to the distributions of mean wind pressure coefficients on M1 in the same wind direction. The locations with higher fluctuating wind pressure coefficients are also with higher mean wind pressure coefficients. In the 0° wind direction, the maximum fluctuating wind pressure is 0.5 that appeared in the leading edges of windward roof and the leeward roof ridge. In the 45° wind direction, due to the formation of conical vortex, the distributions of fluctuating wind pressure appear tapered and nonuniform, and the maximum value is up to 0.7 near the windward roof corner. In the 90° wind direction, the maximum fluctuating wind pressure is 0.5 that appeared in the leading edges of windward roof. For the majority of low-rise buildings with larger rigidity, fluctuating wind pressures on buildings are mainly derived from two factors [7]: (1) natural turbulence or gustiness in the free stream flow and (2) unsteady flow generated by the body itself, by the phenomena such as separations, reattachments, and vortex shedding. Fluctuating wind pressures are mainly generated by the first factor for no separation on windward region. While fluctuating wind pressures on the leading edges of the roof, the roof ridge and the leading edges of the longitudinal walls are mainly generated by the second factor.

Figure 7(b) shows that the distributions of fluctuating wind pressure coefficient are similar to the distributions of mean wind pressure coefficients on M2 in the same wind direction. The locations with higher fluctuating wind pressure coefficients are also with higher mean wind pressure coefficients. The maximum fluctuating wind pressures are 0.6 in the 0° wind direction, 0.8 in the 45° wind direction, and 0.4 in the 90° wind direction, respectively. The fluctuating wind pressure coefficients on M2 are relatively close to those on M1.

## 5. Peak Negative Wind Pressure

### 5.1. Experimental Results

**Figure 8:**
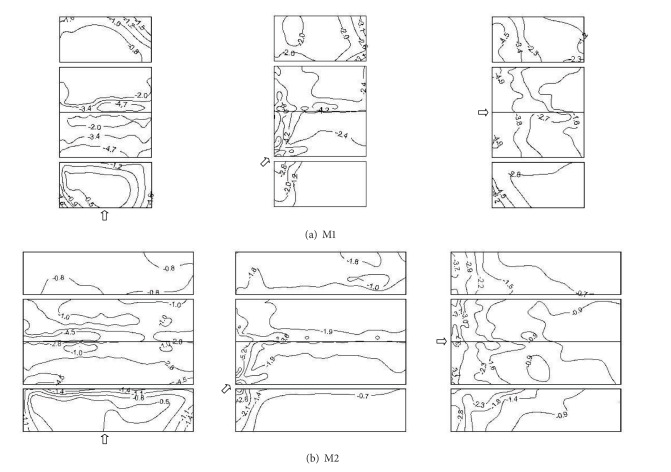
Minimum wind pressure coefficients on M1 and M2.

Postdisaster investigations have provided direct evidence that failure of cladding accounts for much of the initial damage, such as the roof sheathings, the wall sheathings, doors, and windows. It is the presence of the local instantaneous peak negative pressures that primarily affects cladding elements and immediately supporting members [8]. In consideration of the symmetric conditions of the building models, Figure 8 shows the measured results of minimum wind pressure coefficients on M1 and M2 with wind attack angles 0°, 45°, and 90°, respectively.

Figure 8(a) shows that, in the 0° wind direction, the largest absolute values of peak negative wind pressure take place on the edges of windward wall, the leading edges of windward roof, and the leeward roof ridge of M1. In the 45° wind direction, the largest absolute values of peak negative wind pressure occur on the corner and edge of the windward wall, the windward roof corner, and the leeward roof ridge. In the 90° wind direction, the largest absolute values of peak negative wind pressure are present on the windward edges of two longitudinal walls and the leading edges of windward roof.

Figure 8(b) shows the similar distributions of minimum wind pressure coefficients on M2. In the 0° wind direction, the leading edge of windward roof and the leeward roof ridge are the worst loaded regions. In the 45° wind direction, the windward roof corner and the leeward roof ridge are the worst loaded regions. In the 90° wind direction, the leading edge of windward roof is the worst loaded region. It can be noticed that the contour patterns of the minimum wind pressure coefficients are similar to those of the mean wind pressure coefficients shown in Figure 4. This indicates that the largest magnitude peak wind pressures are associated with the largest magnitude mean wind pressures, particularly within separation bubble regions.

### 5.2. Comparative Analysis of Shape Coefficients of Cladding

**Figure 9:**
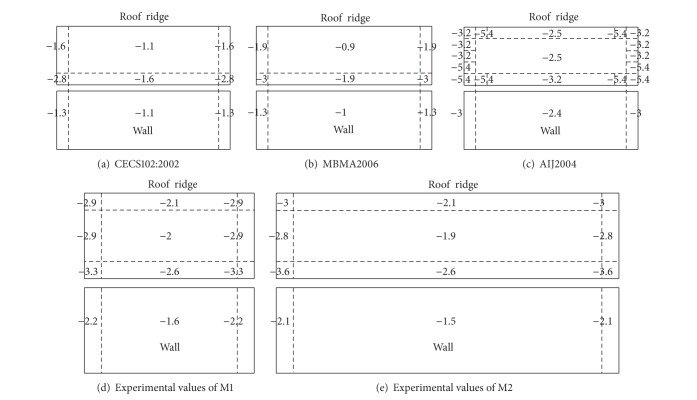
Comparison of experimental values and code values of shape coefficients of cladding.

Larger elements and primary structural systems tend to average the local instantaneous pressures and reduce the dynamic component of the wind load [8]. Design wind pressure of cladding should be the worst peak wind pressure in all kinds of cases [9].  Figure 9 shows the comparison of experimental peak negative pressure coefficients on half of M1 and M2 in consideration of the symmetry of the building irrespective of wind direction and the current code values for the design of cladding, including *Technical Specification for Steed Structure of Light-weight Buildings with Gabled Frames* (CECS102:2002) [10], *Metal Building Systems Manual* (MBMA2006) [11], and *Architectural Institute of Japan Recommendations for Loads on Buildings* (AIJ2004) [12]. The determination method of the edge width from three codes is similar. The edge widths are all 1.8 m. The shape coefficients of cladding in codes take gust effect into account and imply peak negative values irrespective of wind direction. The gust effect factor used in experimental results refers to GB50009-2001. The shape coefficients given by CECS102:2002 refer to MBMA2006. Thus shape coefficients given by CECS102:2002 are closer to MBMA2006. Interior pressure is not considered here.

The presence of high local suctions on the wall edges and roof edges specified in CECS102:2002, MBMA2006, and AIJ2004 is well consistent with experimental results of M1 and M2; however, the experimental values appear higher than code values given by CECS102:2002 and MBMA2006, and relatively close to code values given by AIJ2004. The high peak suctions on roof ridge, due to the flow separation, are not considered in CECS102:2002 and MBMA2006. The shape coefficients from AIJ2004 are more close to experimental results. The edge width specified in code provisions should reveal the most reasonable locations of local high suctions and avoid the spatial average of pressure fluctuations decreasing significantly the effective peak suction. Compared with experimental results, the region division of roof corner and roof ridge from AIJ2004 is more reasonable than those from CECS102:2002 and MBMA2006.

## 6. Proper Orthogonal Decomposition of the Measured Wind Pressure Field

Proper orthogonal decomposition (POD) is a method used to derive the most efficient coordinate system for observing individual phenomena [13]. It can be applied to the analysis of random phenomena, where its efficiency strongly depends on whether a global or a local structural response to wind pressure loads is required. The POD method presents a fast convergence rate for global response, while for local response the effective correlation length of the pressure process must be taken into consideration [14].

The spatial correlation matrix of fluctuating wind pressure based on wind tunnel tests can be decomposed as

(2) $$\begin{array}{c} \left[R_{p}\right]\left[\Phi \right]\;=\;\left[\lambda \right]\left[\Phi \right], \end{array}$$

where [*R*_*p*_] is the spatial correlation matrix of fluctuating wind pressure, [Φ] is the eigenvector matrix, and [*λ*] is the eigenvalue matrix.

The orthogonal eigenvector group obtained from (2) can be regarded as an effective coordinate system of the measured wind pressure field, and the eigenvalues normalized can be regarded as the participation index of corresponding orthogonal eigenvectors to the global fluctuating pressure distribution.

Table 3 shows the eigenvalues, contribution proportions, and cumulative contribution proportions from POD for the pressure field on M1. The eigenvalues derived from POD in this test decrease fast by means of descending order. The 1st mode contributes 27.43%, and the 2nd mode contributes 12.67%. The cumulative contribution proportion up to the 50th mode is about 90% to 93%, while there are a total of 152 modes. This means that about 35% of the modes can reproduce a relatively detailed structure of the wind pressure fluctuations acting on each point of the building surface within an error ranging from 7% to 10%. When wind attack angle is equal to 90°, the contribution of the 1st mode, as well as the cumulated contribution of the first several modes, is significantly smaller in comparison with the results corresponding to the wind attack angle equal to 0° or 45°. 

**Figure 10:**
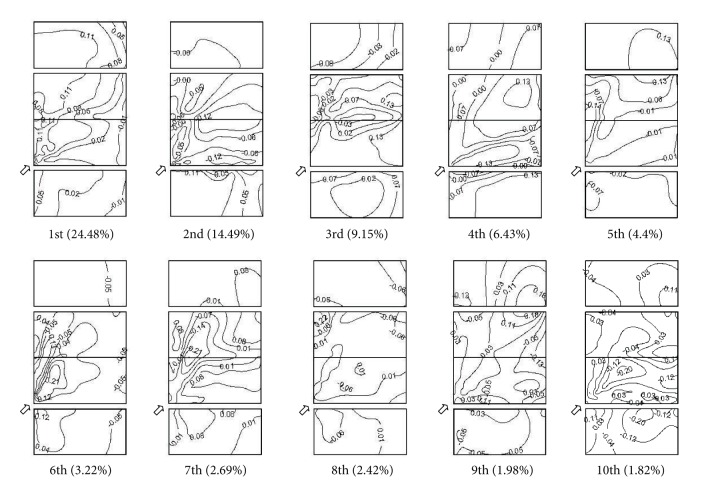
First ten modes from POD on M1 with wind attack angle 45°.

Figure 10 shows the first ten eigenvectors of the measured wind pressure on M1 under wind attack angle 45°. The contribution proportions of each mode to the global wind pressures field are given in the brackets under the contours of corresponding mode. The first several modes give a global description of wind pressure distribution on the model surface, while the other high-order modes just represent local distributions.

## 7. Conclusions

The characteristics of wind pressure acting on the longitudinal walls and roofs of gable roof buildings with different aspect ratio, based on wind tunnel tests, have been described, including mean wind pressure, fluctuating wind pressure, and peak negative wind pressures, and compared with existing results. The characteristics of proper orthogonal decomposition on the building model are obtained based on wind pressure field data. The following conclusions may be made from the study.

1. Local high suctions often occur in the leading edges of longitudinal wall and windward roof, roof corner, and roof ridge, due to the separation of air flow and the formation of conical vortex in oblique wind, which are the severe damaged locations under strong wind. The aspect ratio of building has a certain effect on the mean wind pressure coefficients, and the effect relates to wind attack angle.
2. The experimental shape coefficients appear higher than code values given by CECS102:2002 and MBMA2006 and relatively close to code values given by AIJ2004. Compared with experimental results, the region division of roof corner and roof ridge from AIJ2004 is more reasonable than those from CECS102:2002 and MBMA2006.
3. Due to a good spatial correlation of wind pressure on the surface with edges and corners, the first mode makes a significant larger contribution to a global description of wind pressure distribution, derived from the proper orthogonal decomposition of the measured wind pressure field on gable roof building. The first several modes give a global description of wind pressure distribution on the model surface, while the other high-order modes just represent local distributions.

## Figures and Tables

**Table 1:** Summary of building dimensions.

Model	Full scale building			Model		
	dimensions (m)			dimensions (mm)		
	*H*	*W*	*L*	*H*	*W*	*L*
M1	9	18	18	225	450	450
M2	9	18	36	225	450	900

**Table 2:** Selected full scale gable roof building dimensions of TPU.

Model	Eave height (m)	Width (m)	Length (m)	Roof pitch (°)
TPU1	8	16	16	10
TPU2	8	16	24	10
TPU3	8	16	40	10

**Table 3:** Eigenvalues, contribution proportions, and cumulative contribution proportions for the pressure field on M1.

Mode	0°			45°			90°		
	Eigenvalue	Contribution proportion (%)	Cumulative contribution proportion (%)	Eigenvalue	Contribution proportion (%)	Cumulative contribution proportion (%)	Eigenvalue	Contribution proportion (%)	Cumulative contribution proportion (%)
1st	41.69	27.43	27.43	37.75	24.84	24.84	34.22	22.51	22.51
2nd	19.25	12.67	40.10	22.02	14.49	39.33	14.23	9.36	31.87
3rd	15.01	9.88	49.97	13.90	9.15	48.47	11.12	7.32	39.19
4th	9.76	6.42	56.39	9.77	6.43	54.90	10.60	6.97	46.17
5th	7.16	4.71	61.10	6.69	4.40	59.31	7.40	4.87	51.03
6th	6.47	4.26	65.36	4.89	3.22	62.52	5.79	3.81	54.85
7th	4.11	2.70	68.06	4.09	2.69	65.22	4.93	3.25	58.09
8th	3.70	2.43	70.50	3.68	2.42	67.63	4.18	2.75	60.84
9th	2.49	1.63	72.13	3.01	1.98	69.61	3.52	2.32	63.16
10th	2.37	1.56	73.69	2.76	1.82	71.43	3.40	2.23	65.39
20th	0.98	0.65	82.91	1.11	0.73	82.15	1.37	0.90	77.76
50th	0.27	0.18	93.05	0.29	0.19	93.29	0.39	0.26	90.83
100th	0.09	0.06	98.37	0.08	0.06	98.50	0.12	0.08	97.84
120th	0.06	0.04	99.35	0.05	0.03	99.37	0.07	0.05	99.17
152th	0.00	0.00	100.00	0.01	0.00	100.00	0.00	0.00	100.00
